# The Functional Amyloid Curli Protects *Escherichia coli* against Complement-Mediated Bactericidal Activity

**DOI:** 10.3390/biom8010005

**Published:** 2018-01-24

**Authors:** Steven G. Biesecker, Lauren K. Nicastro, R. Paul Wilson, Çağla Tükel

**Affiliations:** Department of Microbiology and Immunology, School of Medicine, Temple University, Philadelphia, PA 19140, USA; biesecker.steven@gmail.com (S.G.B.); tug03848@temple.edu (L.K.N.); rpwilsonphd@gmail.com (R.P.W.)

**Keywords:** curli, complement, amyloid

## Abstract

*Escherichia coli* strains may be beneficial or pathogenic. Many *E. coli* strains that cause human disease, especially those responsible for bacteremia and sepsis, express virulence factors that impart resistance to the complement system. The bacterial amyloid curli functions in bacterial adherence and enhances the formation of biofilms. Survival of curli-producing parental and curli-deficient mutant *E. coli* in the context of a human complement response was evaluated using an in vivo murine model of bacteremia. Results showed that curli production enhanced *E. coli* survival, which suggests that curli defends against complement-mediated killing. This observation was supported by the results of in vitro assays comparing bacterial survival in human serum. Experiments in which the classical or alternative complement pathways were blocked indicated that the classical pathway is the major contributor to complement activation and that curli inhibits this activity. Our analyses indicate that curli does not appear to play a role in protecting *E. coli* against alternative pathway complement activation. We found that curli increases binding of *E. coli* cells to complement component Complement component 1q (C1q) but does not affect Complement component 3b (C3b) binding. We conclude that curli defends *E. coli* against complement-mediated killing via inhibition of the classical complement pathway.

## 1. Introduction

Due to its ability to adhere to biotic and abiotic surfaces, *Escherichia coli* is capable of successfully inhabiting varied niches including the gastrointestinal tract of vertebrates, plant surfaces, plastic, and steel [1,2]. In immunocompetent individuals, commensal *E. coli* resides within the intestine where it provides the human host with nutrients and protection against pathogenic organisms [3,4,5,6]. Conversely, when coupled with risk factors such as disruption of the intestinal epithelial barrier in patients with diseases like inflammatory bowel disease and acquired immune deficiency syndrome, commensal *E. coli* may cross the intestinal epithelial barrier and cause systemic disease. Left unchecked, a systemic bacterial infection may progress to septic shock, which involves a hyper-inflammatory response that can result in death [7,8]. Expression of various virulence genes by *E. coli* also contributes to a number of pathologic conditions such as infections of the gastrointestinal tract, urinary tract, central nervous system, and bloodstream [9,10]. In the United States alone, over 6.5 million people acquire extra-intestinal *E. coli* infections every year; more than 100,000 cases of *E. coli* infection lead to sepsis [11].

**Figure 1:**
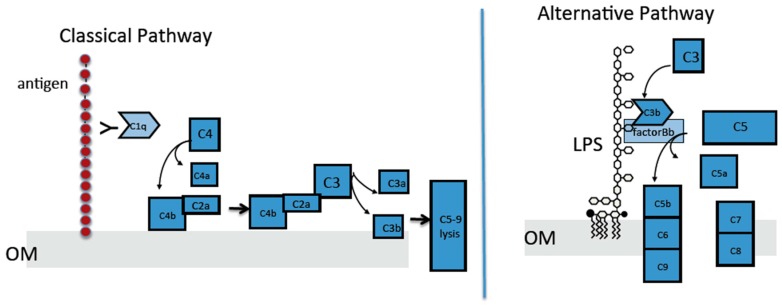
Classical and alternative complement pathway. LPS: Lipopolysaccharide; OM: Outer membrane.

Complement is a system of soluble blood proteins secreted mainly from liver hepatocytes [12] that functions in the opsonization of viruses and bacteria, clearance of immune complexes, and direct killing of bacterial cells through the formation of a membrane attack complex (MAC) [13]. Three distinct pathways have been identified for activation of the complement cascade, which results in bacterial killing: the classical pathway, the lectin pathway, and the alternative pathway. [13]. Interaction between an antibody and a foreign antigen triggers activation of the complement cascade via the classical pathway [13]. The lectin pathway is triggered when mannose-binding lectin or ficolins recognize carbohydrates on foreign surfaces [14]. Finally, the recognition of foreign surfaces by inherently low levels of complement activation initiates the alternative pathway [13] (Figure 1).

Due to its importance in the recognition and clearance of invading microorganisms, bacteria have evolved strategies to evade the complement system. Mechanisms of complement resistance identified in *E. coli* include the modification of lipopolysaccharide (LPS) [15], expression of certain K-antigen capsules [16,17], recruitment of the host regulatory molecules to the outer membrane [18,19], expression of resistance genes encoded by resistance plasmids (R-plasmids) [20,21,22,23], and elimination of immunogens, which inhibits the classical pathway [24]. These mechanisms of complement resistance are often active in pathogenic *E. coli* isolates [25], suggesting their importance during infections.

The formation of a multicellular biofilm provides bacteria with protection against environmental insults, antimicrobial agents, and the host immune response [26,27,28]. As such, there has been much research conducted to understand factors important in biofilm formation. In this search, it was discovered that exopolysaccharides such as cellulose and proteinaceous curli fibrils are expressed in the extracellular matrix of members of the *Enterobacteriaceae* family, including *E. coli*, *Salmonella* spp., *Enterobacter* spp., *Shigella* spp., and *Klebsiella* spp. These extracellular matrix components promote adhesion to biotic and/or abiotic surfaces [1].

Amyloids, such as curli, are proteins possessing a fibrillar, cross-beta sheet structure. Curli fibrils are encoded by the *csgBAC* and *csgDEFG* operons and assembled via a nucleation–precipitation pathway. The *csgA* gene encodes the major subunit of the fibril, CsgA, and the *csgB* gene encodes a minor subunit, CsgB, a nucleator protein [29,30]. Under laboratory growth conditions, curli production is observed only at low temperature and low osmolarity, whereas biogenesis of curli fibrils occurs within the mammalian host at 37 °C [31,32]. In this study, we investigated the protective functions of the curli fibril from *E. coli* against the complement killing system and explored its functions in adherence and biofilm development.

## 2. Results

### 2.1. Characterization of Bacterial Strains

In this study, the MC4100 strain, a K-12 strain commonly used in laboratory research [33], as well as an isogenic mutant that is curli-deficient (*csgBA* mutant, LSR13) were used. MC4100 is a rough strain (lacking LPS O-antigen) that also lacks a K antigen; it was derived from the original K-12 isolate MG1655 [33]. Nissle 1917, an O6:K5:H1 isolate of *E. coli*, and its isogenic curli-deficient mutant (*ΔcsgA*) MDG180 were also utilized in this study.

**Figure 2:**
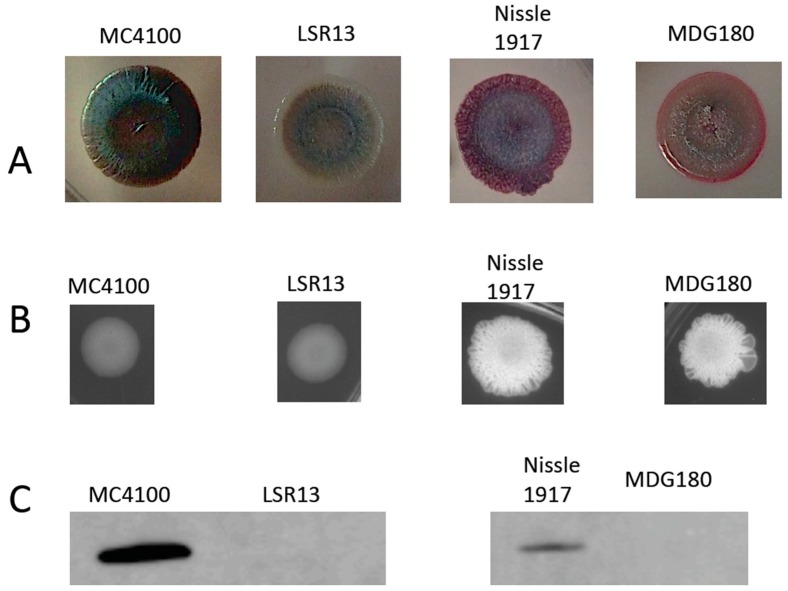
Characterization of bacterial strains used in this study. (**A**) images of representative *Escherichia coli* cells spotted on YESCA plates supplemented with Congo Red (to stain amyloid) and Coomassie Blue (to stain proteins) to characterize curli expression in the MC4100 parental strain (curli +), LSR13 *csgBA* mutant (curli −), wild-type Nissle 1917 strain (curli +), and MDG180 *csgA* mutant (curli −); (**B**) images of *E. coli* cells spotted on Luria Bertani (LB) plates supplemented with 50 μM Calcofluor fluorescent brightener to characterize the expression of cellulose in MC4100 parental strain (curli +), LSR13 *csgBA* mutant (curli −), wild-type Nissle 1917 strain (curli +), and MDG180 *csgA* mutant (curli −); (**C**) Western blot analysis of curli production using anti-CsgA antibodies in MC4100 parental strain (curli +), LSR13 *csgBA* mutant (curli −), wild-type Nissle 1917 strain (curli +), and MDG180 *csgA* mutant (curli −).

To characterize the phenotypes of the bacterial strains used in this study, we first analyzed the ability of cells to bind to Congo Red, previously reported to be an amyloid-specific dye [29]. Strains were grown on (YESCA medium plates to induce curli expression. For staining, medium was supplemented with Congo Red (40 μg/mL) and Coomassie blue (20 μg/mL). MC4100 and LSR13 displayed distinct differences in their ability to bind Congo Red (Figure 2A). MC4100 bound to Congo Red as red colonies were observed, but LSR13 did not and showed white colony morphology. This suggests that the MC4100 strain expressed curli and that the LSR13 strain is curli-deficient as published previously [34]. MDG180 showed pink colony morphology suggesting the presence of cellulose in Nissle 1917 and MDG180 [35]. As the CsgD transcription factor controls cellulose production as well as curli expression [29], we analyzed the expression of cellulose.

To observe the expression of cellulose in *E. coli*, strains were spotted on a Luria Bertani (LB) agar plate supplemented with Calcofluor (Fluorescent Brightener 28). Calcofluor binds to cellulose and fluoresces when exposed to ultraviolet light [36]. *E. coli* strains were grown under curli-inducing conditions and tested for cellulose production. Nissle 1917 and MDG180 produce cellulose while MC4100 and LSR13 did not (Figure 2B).

Confirmation of curli production by the strains used in this study was therefore accomplished through Western blot analysis using an antibody to CsgA. Bacterial strains were tested for curli expression after growth under curli-inducing conditions (i.e., growth on T medium plates at 28 °C for 72 h). Results for the parental strains, MC4100 and Nissle 1917, indicate the presence of CsgA. Alternatively, the curli-deficient mutants, LSR13 and MDG180, did not show a band for CsgA production (Figure 2C). These data demonstrate that the mutant strains of *E. coli* used for these experiments do not express curli.

### 2.2. Curli Enhances Bacterial Cell Survival in an In Vivo Model

**Figure 3:**
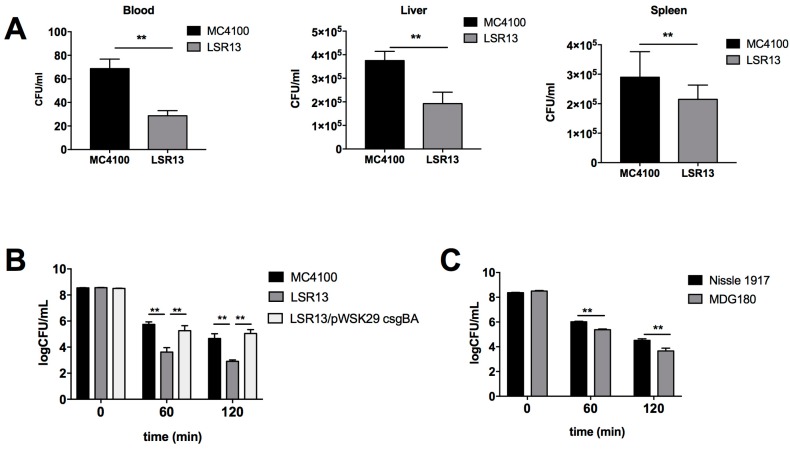
(**A**) CFU/mL of *E. coli* present in the blood, livers, and spleens of mice in a model of *E. coli*-induced sepsis. C57BL/6 mice were infected via tail vein injection of 10^6^ CFU of either MC4100 (curli +, black) or LSR13 (curli −, grey); (**B**) bacterial numbers were enumerated for *E. coli* MC4100 (curli +, black), LSR13 (*ΔcsgBA*, curli −, grey), and CT183 (complemented, curli +, white) after 0, 60 or 120 min of exposure to human complement serum; (**C**) analysis of *E. coli* strains Nissle 1917 (curli +, black) and MDG180 (curli −, grey) in their sensitivity to human complement serum over 120 min. Asterisks indicate significance (*p* < 0.05).

Curli fibrils have previously been associated with a pro-inflammatory immune response to infections with *S. enterica* serovar Typhimurium and *E. coli* [37,38,39,40,41]. In order to investigate whether curli has other effects during infection, a murine model of *E. coli*-induced sepsis was used to determine if production of the curli fibril impacts bacterial survival during systemic infections. For this experiment, groups of C57BL/6 mice were injected with 10^6^ colony forming units (CFU) of either MC4100 or LSR13 *E. coli* strains through the tail vein. After 2 h or 8 h, the mice were sacrificed and blood samples, as well as liver and spleen samples were obtained, and bacterial numbers were quantified. After 2 h, mice infected with the curli-producing MC4100 strain had significantly more bacteria present in their blood, liver, and spleen than mice infected with the curli-deficient LSR13 strain (Figure 3A). When the infection period was extended to 8 h, there was no significant difference [42]. These data suggest that curli provides *E. coli* with protection from early host innate immune defense in the blood.

### 2.3. Curli Inhibits Complement-Mediated Killing of Bacterial Cells In Vitro

As curli expression increased survival of *E. coli* in the blood early in infection, we hypothesized that curli provides resistance to the complement system, a major host defense system [13]. To evaluate this hypothesis, we utilized an in vitro model of complement-mediated killing. In this serum sensitivity assay, significantly higher numbers of MC4100 cells survived after 60 and 120 min of complement exposure than LSR13 cells (Figure 3B). In order to confirm that this effect was due to the presence of curli, a strain of LSR13 complemented with *csgBA* genes was also tested. Curli production restored the survival of bacteria to the levels observed for the parental MC4100 strain (Figure 3B). Curli expression was also protective in the background of the Nissle 1917 strain (Figure 3C), indicating that this effect is not specific to the MC4100 strain. These data indicate that curli protects *E. coli* cells from the complement system.

### 2.4. The Classical Complement Pathway Is Inhibited by Curli

**Figure 4:**
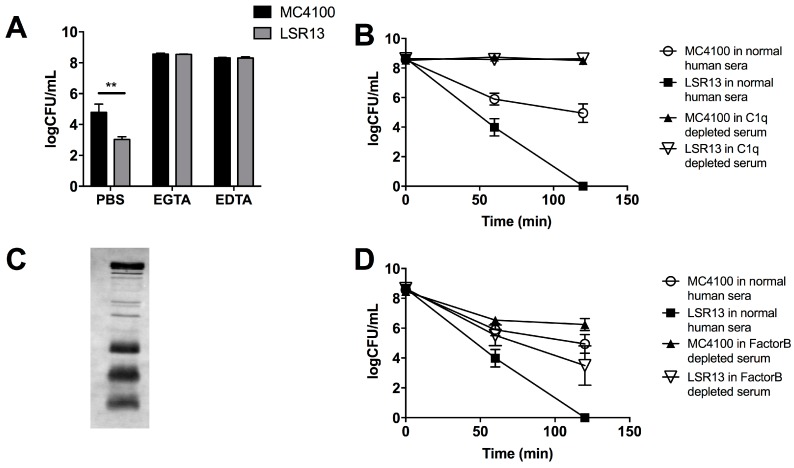
(**A**) analysis of the effect of complement on *E. coli* strains MC4100 (curli +, black) and LSR13 (curli −, grey) when complement pathways are blocked. Control samples were prepared in PBS. All complement pathways were blocked by addition of EDTA to bacterial cultures to a final concentration of 10 mM. The classical complement pathway was blocked selectively through the use of 10 mM EGTA and 5 mM MgCl_2_; (**B**) time course analysis of viability of *E. coli* strains MC4100 (curli +) and LSR13 (curli −) in Complement component 1q (C1q)-depleted serum and normal serum; (**C**) Western blot analysis for the presence of *E. coli*-reactive antibodies in human serum; (**D**) time course analysis of viability of *E. coli* strains MC4100 (curli +) and LSR13 (curli −) in human serum depleted of Factor B.

To identify the mechanism by which curli provides protection to *E. coli* against complement, specific pathways of complement activation were blocked and effects on bacterial survival were evaluated. Two approaches were used. The first approach utilized a solution of the magnesium salt of ethylene glycol-bis(β-aminoethyl ether)-*N*,*N*,*N*′,*N*′-tetraacetic acid (MgEGTA), which specifically blocks the classical complement pathway [43]. In the presence of MgEGTA, there was no difference in survival between the parental and curli-deficient strains in response to serum (Figure 4A). When all three pathways were inactivated using ethylenediaminetetraacetic acid (EDTA), no differences were observed between the parental and curli-deficient strains in response to serum (Figure 4A). This suggests that the classical pathway is inhibited by curli.

As a second approach, we utilized Complement component 1q (C1q)-depleted serum; in this serum, the classical pathway is inactive, but the alternative and lectin pathways are unaffected. Parental and curli-deficient strains were incubated either in normal human sera or C1q depleted sera. LSR13 was cleared from the serum after a 120-min, whereas viability of MC4100 was only partially affected (Figure 4B). These results suggest that complement-mediated killing of *E. coli* is dependent upon the activity of the classical pathway and that curli inhibits this pathway.

We next performed a Western blot analysis to determine whether antibodies specific to *E. coli* were present in human serum used in complement-mediated killing assays. *E. coli* cells were lysed, and extracts were subjected to electrophoresis on a sodium dodecyl sulfate polyacrylamide gel electrophoresis (SDS-PAGE) gel. Proteins were transferred to Polyvinylidene fluoride (PVDF) membrane, and membranes were incubated with human serum. These experiments revealed that anti-*E. coli* antibodies are present in the commercially available human serum (Figure 4C).

### 2.5. The Alternative Complement Pathway Is Not Influenced by Curli

To investigate whether the alternative complement pathway is affected by the presence of curli, we used serum selectively depleted of Factor B, a component necessary for alternative pathway activation [13]. When exposed to Factor B-depleted serum, MC4100 survived significantly better than the LSR13 curli-deficient mutant; however, the level of killing by the Factor B-depleted sera was less than that of normal human serum for both strains (Figure 4D). These data suggest that the alternative complement pathway is not the main contributor to complement-mediated killing of *E. coli*. The significant difference in viability of *E. coli* between the parental and mutant strains in the Factor-B-depleted serum indicates that curli provides protection against non-alternative pathway complement killing.

### 2.6. E. coli Cells Bind C1q but Not C3b

**Figure 5:**
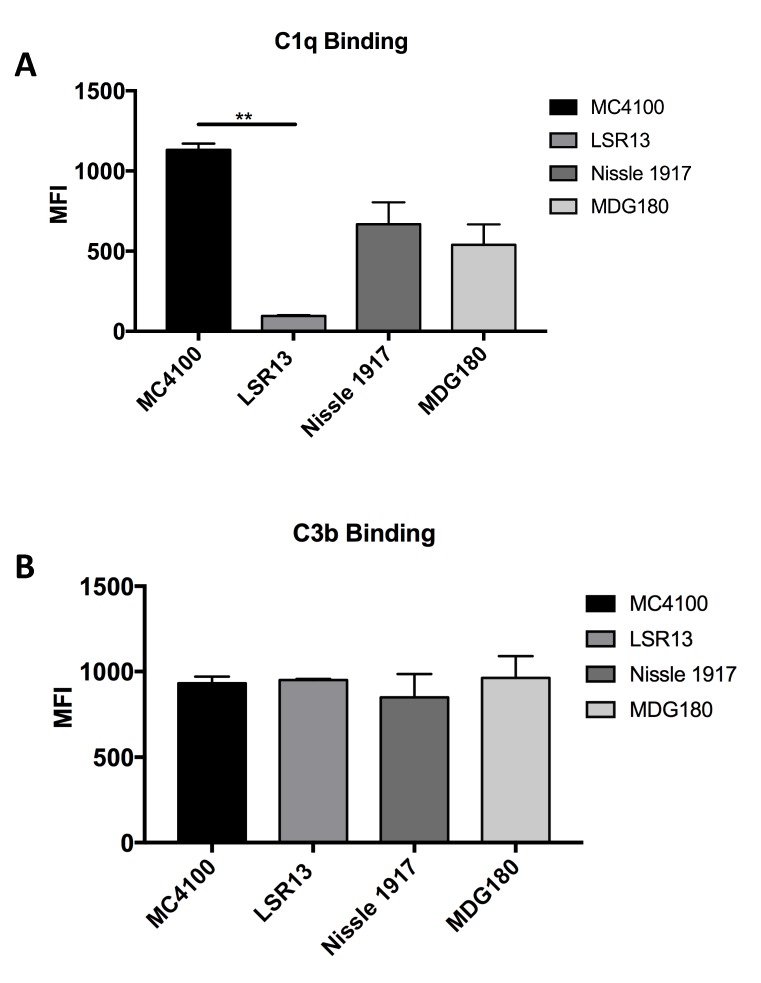
Binding of *E. coli* strains MC4100 (curli +), LSR13 (curli −), Nissle 1917 (curli +), and MDG180 (curli −) to (**A**) C1q and (**B**) C3b as measured by flow cytometry in mean fluorescence intensity units (MFI). Each graph displays the combined results of two replicates. Geometric means for each sample were averaged to obtain depicted values. Asterisks indicate significance (*p* < 0.05).

In order to investigate mechanisms by which curli protects *E. coli* from complement-mediated killing, we tested whether curli alters the deposition of certain complement components on the bacterial cells. Previous studies have provided evidence that amyloids, such as those involved in Alzheimer’s Disease, bind to C1q [44]. As C1q is a necessary component for activation of the classical complement pathway, we analyzed the effect of curli on the binding of C1q by *E. coli*. The protein affect Complement component 3b (C3b) is important in both the lytic complement pathway and opsonophagocytosis [43]; therefore, we also evaluated binding of C3b to the bacterial cell surface. Using flow cytometry, we analyzed the binding of these complement components in curli-producing and curli-deficient *E. coli*. Significantly higher levels of C1q bound to MC4100 cells than to the curli-deficient strain LSR13 (Figure 5A). There was not a significant difference in binding of C1q to Nissle 1917 and its curli-deficient mutant (Figure 5A). Curli had no effect on binding of C3b: the MC4100 and Nissle 1917 parental strains and curli-deficient strains bound C3b to similar extents (Figure 5B). These data indicate that curli increases binding of certain strains of *E. coli* cells to complement component C1q but does not affect C3b binding.

## 3. Discussion

Bacteria efficiently colonize the mucosal surfaces of the human body. More than 100 trillion bacteria colonize the gastrointestinal tract alone [6,45,46]. In order to colonize, survive, and compete for nutrients on mucosal surfaces, bacteria form multicellular communities known as biofilms. Biofilms provide protection against environmental insults, antimicrobial agents, and the host immune response [26,27,28]. Exopolymers, such as cellulose and curli, expressed by many species of the *Enterobacteriaceae* family, including *E. coli*, *Salmonella* ssp., *Enterobacter* spp., *Shigella* spp., and *Klebsiella* spp. influence biofilm development [1].

Defense against the complement system imparts a great advantage to both pathogenic and non-pathogenic bacteria during survival on mucosal surfaces [15,17,25]. Multiple mechanisms have been identified through which bacteria evade killing by the complement system. Some pathogenic Gram-negative bacteria block the formation of the MAC complex on the membrane through extended O-antigen, one of the major integral components of LPS. For instance, the *Salmonella minnesota* membrane is resistant to insertion of the MAC complex due to the hydrophobic outer membrane [15]. In addition, some capsule serotypes inhibit opsonization by complement component C3b [16]. By incorporating sialic acid in the K capsule, Factor H can bind to increase the degradation of complement components C3b and C4b [13,47,48]. CD59 (also known as protectin) is a defender of human cells against lysis by the complement system; CD59 is recruited by some *E. coli* cells enabling them to evade lysis by complement [18]. Although previous studies have demonstrated that biofilms provide protection against complement-mediated phagocyte killing by blocking engulfment [49], the components of biofilm that directly inhibit complement killing remained unknown. For instance, it has been determined that *Staphylococcus epidermidis* biofilms inhibit the deposition of C3b on the bacterial surface, providing protection from complement-mediated killing by polymorphonuclear leukocytes [50]; however, the components of the *S. epidermidis* biofilm that inhibit C3b deposition have yet to be identified.

Here, we demonstrate that curli, an important component of enteric biofilms, protects *E. coli* from complement-mediated killing by providing resistance against the antibody-mediated classical complement pathway. We showed that curli expression by two different *E. coli* strains provides protection in an in vivo model of systemic infection as well as in vitro serum sensitivity assays. Interestingly, the survival advantage demonstrated in vivo at 2 h post-infection disappeared as the infection progressed (Figure 3). It is important to note that the colonization status of these mice with *E. coli* or the level of antibodies against *E. coli* was not determined at the time of infection. However, the mice generated by the vendor used in these studies are known to be clear of *E. coli* colonization. Therefore, it is possible that the previous exposure of mice to *E. coli* may dramatically enhance the phenotype we observed, as the generation of *E. coli* specific antibodies would increase the classical complement pathway activity. Furthermore, other host defense systems likely inhibit the proliferation of these non-pathogenic strains, regardless of curli production. In pathogenic bacteria, however, such defenses may be less effective due to the presence of other virulence factors. Curli may provide a first line of defense against the rapid complement response, allowing the bacteria to establish themselves before delayed host responses are triggered. Our data indicate that *E. coli*-reactive antibodies were present in the human serum obtained from a commercial supplier that was used in our study. It is important to note that *E. coli*-reactive antibodies were observed at much lower levels in serum obtained from another commercial supplier, Sigma-Aldrich (St. Louis, MO, USA). Although serum from both suppliers showed similar patterns for killing *E. coli* and its isogenic curli mutant, we also observed that serum from Quidel (San Diego, CA, USA) was tenfold more effective in killing bacteria [42]. Pathogenic *E. coli* strains cause many common bacterial infections including cholecystitis, bacteremia, cholangitis, urinary tract infections, traveler’s diarrhea, and other clinical infections such as neonatal meningitis and pneumonia [11,51]. Thus, it is difficult to estimate the incidence of *E. coli* infections. Infections by other organisms may allow *E. coli* to opportunistically cross the epithelial barrier resulting in an adaptive immune response [52]. In addition to infections, bacterial antigens sampled by resident dendritic cells may be involved in the generation of specific antibodies [53]. Nevertheless, the presence of curli-specific antibodies in the commercially supplied human serum remains unclear.

Serum sensitivity assays using MC4100 and Nissle 1917 confirmed that our observations were not strain specific. Whereas Nissle 1917 results were similar to that of MC4100, the curli mutant strains differed in their survival. One reason for this could be that factors in addition to curli influence survival of bacteria confronted with the complement response. It was previously shown that the K5 capsular antigen, present in Nissle 1917, is a poor immunogen for generation of classical pathway-activating antibodies [54]. Another potential difference could be that Nissle 1917 produces cellulose, whereas MC4100 does not, and cellulose may be masking the epitopes required for C1q binding and the activation of classical pathway. Comparison with a Nissle 1917 and its isogenic cellulose mutant would confirm whether cellulose has an effect on bacterial survival.

The binding of antigen-specific antibodies, followed by the attachment and activation of the C1 complex, are critical events in the initiation of the classical complement pathway. Recent studies demonstrated that beta-amyloid, the major constituent of senile plaques, binds to C1q and activates the classical complement pathway, suggesting that C1q activation contributes to tissue damage in Alzheimer’s disease [44,55,56]. Subsequently, it was demonstrated that beta-amyloid also binds to a fluid-phase complement inhibitor C4b-binding protein (C4bp) [57]. The mechanism by which complement activation by beta-amyloid and inhibition through C4bp contributes to Alzheimer’s disease remains unknown. Conservation of the quaternary structure of amyloid may account for similar functional properties among amyloids; human and bacterial amyloids bind fibronectin [58,59] and laminin [60,61] and activate fibronectin and plasminogen [61,62,63].

Consistent with the beta-amyloid-C1q interaction [44,55,56], we demonstrated that bacteria that express curli bound more C1q than their curli-deficient counterparts (Figure 5A). Although parental bacterial strains recruited more C1q to their surfaces, these bacteria were more resistant to killing by the classical complement pathway than curli-deficient strains, suggesting that the subsequent steps in the classical pathways were abrogated by the presence of curli fibrils (Figure 1). Curli fibrils may interfere with the assembly of the MAC on the bacterial membrane by providing a physical barrier. This could eventually result in the shedding or trapping of the MAC in amyloid fibrils. This idea is consistent with the fact that the C3b deposition is similar between the bacteria that has curli or not (Figure 5B). Another possible mechanism for the complement resistance by curli could be through binding of C4bp, which is bound by beta-amyloid to inhibit the classical pathway [57]. This mechanism would be very similar to the resistance provided by outer membrane protein (OmpA) [19].

In summary, this study has demonstrated that the curli amyloid fibril, an important component of bacterial biofilm, protects *E. coli* from complement-mediated killing. Curli production enhanced *E. coli* survival in vivo and in vitro in the presence of complement-containing human serum. Our experiments indicated that the classical pathway is the major contributor to complement activation by *E. coli* and that curli inhibits this activity.

## 4. Materials and Methods

### 4.1. Bacterial Strains and Growth Conditions

For this study, two *E. coli* strains were used: MC4100 and Nissle 1917. Isogenic curli-deficient strains for each of these parental strains were obtained to examine the effect of curli expression. The *csgBA* mutant LSR13 and the parental MC4100 strain were kindly provided by Dr. Scott Hultgren (Washington University, St. Louis, MO, USA). Nissle 1917 and its *csgA* mutant MDG180 (Kan^R^) were kindly provided by Dr. Mark Goulian (University of Pennsylvania, Philadelphia, PA, USA). The strain CT183 (Carb^R^) was constructed by complementing the LSR13 strain with the pWSK29 plasmid encoding the *csgBA* genes [41]. For optimal growth, strains were inoculated into 5 mL of LB broth and incubated overnight at 37 °C with 200 rpm agitation in a platform shaker (VWR, Radnor, PA, USA). To induce curli expression, strains were grown on T medium plates at 28 °C for 72 h as previously described [39,64]. Expression of curli was confirmed through Western blot analysis using anti-CsgA antibodies [65]. For visual characterization of curli expression, bacterial strains were grown at 28 °C for 72 h on YESCA agar plates supplemented with 40 μg/mL Congo Red and 20 μg/mL Coomassie Blue [66]. For visual analysis of cellulose production, bacterial strains were grown under curli-inducing conditions on YESCA plates supplemented with 50 μM Calcofluor (Fluorescent Brightener 28, Sigma-Aldrich, St. Louis, MO, USA) [67]. Bacteria were then exposed to ultraviolet light and imaged using a Universal Hood II Gel Imager (Bio-Rad, Hong Kong, China).

### 4.2. Serum Sensitivity Assays

Serum sensitivity assays were carried out as described by Wilson and colleagues [43]. Briefly, bacterial strains were grown under curli-inducing conditions described above. Bacteria were then scraped off the plates and suspended in phosphate buffered saline (PBS). Optical density of the solutions was measured at 600 nm using a spectrophotometer (Thermo Scientific, Waltham, MA, USA; Genesys 10S Series) and adjusted to an optical density of 0.700. 10^8^ CFU of bacteria was added into PBS that contains 10% Normal Human Complement Standard (Quidel). Mixtures were incubated at 37 °C and sampled at 0, 60, and 120 min. Bacterial suspensions were diluted 1:10 in PBS and plated on LB agar plates with appropriate antibiotics. Carbenicillin (100 μg/mL final concentration) was used for plating of LSR13/pWSK29 *csgBA.* Kanamycin (100 μg/mL final concentration) was used for MDG180. Plates were incubated overnight at 37 °C. Resultant bacterial CFU were counted, and CFU per mL of bacterial suspension were calculated. All serum sensitivity assays were repeated at least three times.

### 4.3. Inhibition of Complement Pathways

To inhibit all three complement pathways, a solution of EDTA was used at a final concentration of 10 mM [68]. To specifically block the classical complement pathway, EGTA and MgCl_2_ were added to a final concentration of 10 mM and 5 mM, respectively [68]. Bacterial strains were grown for 72 h on T medium plates at 28 °C. Bacteria were resuspended in PBS and optical densities at 600 nm were adjusted to 0.700. A mixture of 10% Normal Human Complement Standard and 10% bacterial suspension was prepared in PBS containing 10 mM EDTA or 10 mM EGTA with 5 mM MgCl_2_ [68]. C1q-depleted serum (Quidel) was used as the source of serum in experiments in which the classical pathway was inhibited. For specific inhibition of the alternative complement pathway, Factor B depleted serum (Quidel) was used. Mixtures were incubated at 37 °C for 0, 60, and 120 min. Bacterial suspensions were diluted 1:10 in PBS and plated on LB agar plates with appropriate antibiotics. Plates were incubated overnight at 37 °C. CFU and CFU per mL of bacterial suspension were determined. Assays were repeated at least three times.

### 4.4. Western Blot

For detection of *E. coli*-specific antibodies in human serum, *E. coli* Nissle 1917 and MC4100 lysates were prepared by suspending bacteria in PBS and boiling with SDS-PAGE loading buffer. Lysates were then electrophoresed on a 12% SDS-polyacrylamide gel. The presence and quantity of protein in the samples was confirmed through staining with Coomassie Blue and detection with the ODYSSEY infrared imaging system (Li-Cor Biosciences, Lincoln, NE, USA) at 700 nm. Proteins were transferred to a PVDF membrane using a Semi-Dry Transfer Cell (Bio-Rad) for 1 h (15 V, 0.15 A). Membranes were then incubated for 1 h in a 1:1000 dilution of Normal Human Complement Standard serum in blocking buffer (1× PBS containing 5% non-fat dry milk and 0.05% Tween 20). Membranes were then washed three times with blocking buffer. For the detection of human sera, membranes were incubated for 1 h with a 1:10,000 dilution of Li-Cor ODYSSEY goat anti-human IRDye 800CW secondary antibody in blocking buffer. Membranes were washed three times in blocking buffer, followed by three washes in PBS. Detection was done through Li-Cor ODYSSEY detection at 800 nm. For analysis of rabbit complement sera, Li-Cor ODYSSEY goat anti-rabbit IRDye 800CW antibody was used as the secondary antibody.

To detect curli expression by Western blot, samples were prepared by formic acid treatment as described previously [39,64]. Briefly, *E. coli* strains were grown under curli-inducing conditions. Bacteria were washed in PBS and then resuspended in 90% formic acid and snap frozen in an ethanol-dry ice bath. Next, formic acid was removed using a Savant Speed Vac Concentrator (Thermo Scientific) for 1.5 h. Samples were then resuspended in PBS and SDS-PAGE loading buffer. Electrophoresis and membrane transfer were carried out as described above. Rabbit anti-CsgA antibodies (1:1000 dilution) were used as primary antibodies [65]. Secondary antibody incubation was accomplished through use of a 1:10,000 dilution of Li-Cor ODYSSEY goat anti-rabbit IRDye 800CW antibody.

### 4.5. C1q and C3 Binding Assay

Detection of C1q binding was done as described by Wilson et al. [43]. *E. coli* strains were grown under curli-inducing conditions for 72 h. Bacteria were resuspended in PBS containing 10% C5-depleted sera (Quidel) and incubated at 37 °C for 30 min. Cells were washed twice with PBS and killed by incubation in PBS containing 0.1% sodium azide (*w*/*v*) at room temperature for 20 min. Samples were then washed twice with PBS. To detect C1q binding, samples were resuspended in PBS and Fluorescein isothiocyanate (FITC)-conjugated goat IgG fraction to mouse complement C1q (1:250 dilution) was added. Samples were incubated for 1 h in the dark. Samples were then washed three times with PBS and analyzed via flow cytometry.

For C1q binding assays, samples were resuspended in PBS containing 2% goat serum and incubated at room temperature for 30 min. A 1:250 dilution of rabbit polyclonal antibody to C1q (Abcam, Cambridge, United Kingdom) was then added. Samples were then incubated for 1 h at room temperature. Cells were washed three times in PBS and then resuspended in PBS containing a 1:250 dilution of FITC-conjugated AffiniPure goat anti-rabbit IgG antibody. Samples were then incubated in the dark at room temperature for 1 h. After washing three times in PBS, C1q binding was detected using the FACSCanto Flow Cytometer (BD, Franklin Lakes, NJ, USA). Data were analyzed and mean fluorescence intensity for samples was calculated using FlowJo software (Flowjo LLC, Ashland, OR, USA). Each binding assay was performed in triplicate or greater.

### 4.6. Animal Experiments

Mouse experiments in this study utilized six- to eight-week-old female C57BL/6 mice purchased from Jackson Laboratories (Bar Harbor, ME, USA). *E. coli* strains MC4100 (parental) and LSR13 (*ΔcsgBA*, mutant) were prepared under curli-inducing conditions, as described above, for 72 h. Bacteria were scraped from the plates and suspended in PBS. Optical density of the bacterial solutions was measured and diluted to an absorbance of 0.700 at 600 nm. This suspension was then diluted 1:100 in PBS to result in 10^7^ CFU/mL bacteria. Mice were given 100 μL of the bacterial suspension via intraperitoneal injection. One group of three mice was injected with MC4100, whereas another group of three was injected with LSR13. At 2 h post-infection, mice were sacrificed, and blood was collected via cardiac puncture. Blood samples were collected in tubes containing 5 μL of 19.2 ng/μL heparin. Bacterial numbers in blood were determined after overnight incubation on LB plates at 37 °C, and CFU/mL was calculated. Mouse experiments were approved by Temple University’s Institutional Animal Care and Use Committee under protocol 3328. 

### 4.7. Statistical Analysis

For the analysis of bacterial survival, measurements of CFU/mL were transformed logarithmically. Variance from multiple experiments was determined by calculation of mean values and standard deviations. Statistical significance of bacterial survival assays was determined through the use of an unpaired Student’s *t*-test (*p* < 0.05).
